# Laboratory Evaluation on the Performance Degradation of Styrene-Butadiene-Styrene-Modified Asphalt Mixture Reinforced with Basalt Fiber under Freeze–Thaw Cycles

**DOI:** 10.3390/polym12051092

**Published:** 2020-05-11

**Authors:** Yongchun Cheng, He Li, Wensheng Wang, Liding Li, Haitao Wang

**Affiliations:** 1 College of Transportation, Jilin University, Changchun 130025, China; chengyc@jlu.edu.cn (Y.C.); lihe326532558@163.com (H.L.); lild17@mails.jlu.edu.cn (L.L); wht19@mails.jlu.edu.cn (H.W.); 2 Ingram School of Engineering, Texas State University, San Marcos, TX 78666, USA

**Keywords:** asphalt mixture, basalt fiber, freeze–thaw cycle, performance degradation

## Abstract

This paper aims at the freeze–thaw (F-T) cycles resistance of styrene-butadiene-styrene (SBS) modified asphalt mixture reinforced with basalt fiber in order to explore the performance evaluation and prediction of asphalt mixtures at seasonal frozen regions. Asphalt was firstly modified by the common SBS and then SBS-modified stone mastic asphalt (SMA) specimens with basalt fiber were prepared by using Superpave gyratory compaction (SGC) method. Next, asphalt mixture specimens processed by 0–21 F-T cycles were adopted for the high-temperature compression test, low-temperature splitting test and indirect tensile stiffness modulus test. Meanwhile, a three-dimensional model of F-T damage evolution of the mixtures was also established based on the reliability and damage theory. The test results showed that the loss rates of mechanical strength increased rapidly, and then gradually flattened; however, these indications changed significantly after 15–18 F-T cycles. In addition, the exponential function could reflect the variation trend of the mechanical performances with F-T cycles to a certain degree. The damage evolution and prediction model based on the reliability and damage theory can be established to analyze the internal degradation law better.

## 1. Introduction

Flexible pavement has been widely used in the construction of high-grade highways due to its advantages such as good driving performance, high comfort, low noise, and convenient construction and maintenance [1,2]. In the long-term service process of asphalt pavement, the complex and diverse service environment as well as increasing traffic and load would lead to more and more pavement damages, causing damage and greatly shortening the service life [3,4]. Especially in northern China and other seasonal frozen areas, due to severe climate changes and traffic load, asphalt pavement will be severely damaged and show accelerated deterioration [5,6,7].

As it is known that basalt fiber is produced from natural basalt material after melting at high temperatures, and as a new type of environmentally friendly and high-performance inorganic fiber material, basalt fiber has the advantages of high strength, low water absorption, acid and alkali resistance, high temperature resistance, and environmental protection [8,9,10,11]. Many researchers have carried out a large number of related experimental studies on basalt fiber-modified asphalt mixtures. Moreover, basalt fibers have been gradually used in asphalt pavements to replace polyester fibers and lignin fibers. It was also evident that the strong affinity between basalt fiber and asphalt can improve its road performance and effectively improve the durability of asphalt mixtures [12,13,14].

With the development of basalt fiber production technology, Chinese road researchers have learned from foreign application experience about basalt fiber and conducted a large number of laboratory tests and engineering application related to basalt fiber-modified asphalt [15,16,17,18]. Xiong et al. [19] conducted a comparative study on the improvement mechanism of various fiber-modified asphalt mortars, and evaluated the water absorption, oil absorption, and thermal stability of fibers. The cone penetration and dynamic shear rheology tests were used to evaluate the shear strength, rheological properties and rutting resistance of various fiber-modified asphalt. Miao et al. [20] studied the fiber-reinforced asphalt mortar through interfacial adhesion properties, and used a variety of fibers and asphalt to measure the surface energy and shear strength of different asphalt mortars, respectively. The test results showed that the addition of fibers could significantly improve the shear strength of asphalt mortar. In contrast, basalt fibers have the best reinforcement effect. Through experimental research on a variety of fiber-modified asphalt mortars, it is clear that basalt fibers have a better improvement effect. Then, researchers conducted a large number of laboratory studies on the effects of parameters such as basalt fiber content and length on the performance of asphalt mortar. Wang et al. [21] studied the effect of basalt fibers on the low-temperature properties of asphalt mortar, and tested and evaluated basalt fiber-modified asphalt mortar using tensile tests and fatigue tests. The test results show that basalt fiber can improve the tensile strength of asphalt mortar and significantly improve its fatigue resistance. Kathari et al. [22] studied the effect of basalt fiber content on the high-temperature performance of asphalt mortar, and used a scanning electron microscope to conduct a microscopic analysis of basalt fiber. The test results showed that basalt fiber-modified asphalt mortar has good resistance to deformation under high temperature conditions, and it is considered that the improvement effect was better when the basalt fiber content is 1%.

In order to further utilize basalt fiber to improve the mechanical properties of asphalt mixtures, road researchers have also made a lot of efforts and conducted many related tests and verifications. Aiming at the high temperature performance of asphalt mixture with basalt fiber, Wu et al. [23] systematically investigated the effect of basalt fiber on the high-temperature stability of the mixtures. The dynamic modulus test, dynamic creep test and rutting test were used to evaluate the test results. It was shown that adding basalt fibers can obviously improve the high temperature resistance to deformation of the mixtures. Regarding the low-temperature performance of the mixture, Shen et al. [24,25] used tensile and bending tests to explore the effect of basalt fiber length and content on the low-temperature crack resistance of asphalt mortar. Then the correlation analysis between asphalt mortar and mixture was used for the low-temperature crack resistance of basalt fiber-modified asphalt mixtures. As for water damage of basalt fiber reinforced asphalt mixture, Zhang et al. [26] conducted a systematic study on the water stability of various additive-modified asphalt mixtures under dry and wet cycling conditions. Comprehensive analysis was made using immersion Marshall, freeze–thaw splitting, hydrodynamic pressure scour splitting, and immersion Hamburg rutting test. The results illustrated that basalt fiber has the best effect on improving the water stability of asphalt mixtures, and could significantly improve the water loss resistance of asphalt pavements in high humidity areas. Wang et al. [27,28] studied the F-T damage of Marshall AC-13 gradation with basalt fiber of 6-mm length by using ordinary mechanical performances and then used the developed logistic damage model to predict the damage evolution.

In view of the above situation, this paper focuses on the freeze–thaw (F-T) cycles resistance of SBS modified asphalt mixture reinforced with basalt fiber in order to explore the performance evaluation and prediction of asphalt mixtures at seasonal frozen regions. Asphalt mixture specimens prepared by SGC were adopted and processed by 0-21 F-T cycles for the high-temperature compression test, low-temperature splitting test and indirect tensile stiffness modulus test. Then, a three-dimensional model of F-T damage evolution of the mixtures would be established by using the reliability and damage theory in order to study the damage evolution.

## 2. Materials and Methods

### 2.1. Raw Materials and Specimen Preparations

The selected asphalt is SBS modified asphalt for this study, which was produced by Zhonghai asphalt Co., Ltd. (Yingkou, China). The coarse and fine aggregates in this paper are crushed basalt, produced from Jiutai City, Jilin Province. The filler used in this paper is limestone power, which was from Siping City, Jilin Province. In addition, eco-friendly basalt fiber was used to reinforce asphalt mixture in this paper. The technical properties of all above materials refer to the previous studies [27,29].

**Figure 1:**
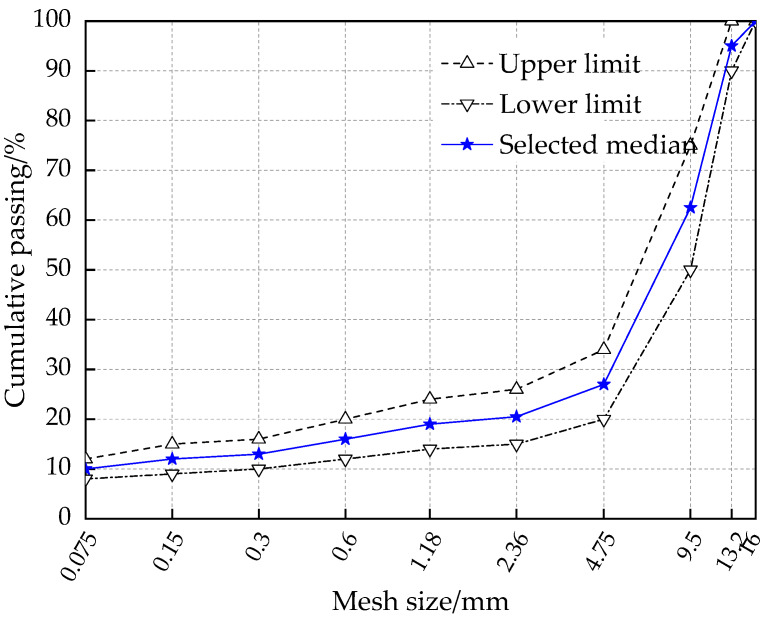
Gradation of SMA-13 in this study.

The stone mastic asphalt (SMA) is a commonly used asphalt mixture type in China, and SMA is extensively adopted for highways. Following the standard [30], the median gradation of SMA-13 was selected in this paper. The corresponding gradation range is plotted in Figure 1. The asphalt mixture specimens were prepared through Superpave gyratory compaction (SGC) method, and the detailed procedure could be investigated in the previous study [29].

### 2.2. Experimental Procedure and Methods

#### 2.2.1. Experimental Procedure

**Figure 2:**
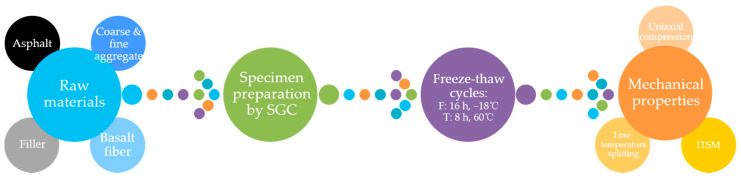
Flowchart of the experimental procedure used in this paper.

In this paper, asphalt mixture specimens were firstly prepared by using SGC method. The second step in the experimental procedure focused on conducting the freeze–thaw (F-T) cycles for asphalt mixture specimens, in which these specimens were subjected to 0, 3, 6, 9, 12, 15, 18, 21 F-T cycles, respectively. Before F-T cycle processing, the specimens should be sealed separately with a plastic bag, and then poured 15 mL of water in bags. Then immediately put the test specimens into the refrigerator. Each F-T cycle includes freezing the test specimens in an −18 °C refrigerator for 16 h and then thawing them in 25 °C water for 8 h. As for the mechanical properties, high-temperature uniaxial compression strength, low-temperature splitting strength and indirect tensile stiffness modulus (ITSM) were chosen as the mechanical indexes to access the mechanical degradation of asphalt mixture under F-T cycles. The flowchart of the experimental procedure is shown in Figure 2.

#### 2.2.2. Experimental Methods

##### High-Temperature Uniaxial Compression Test

**Figure 3:**
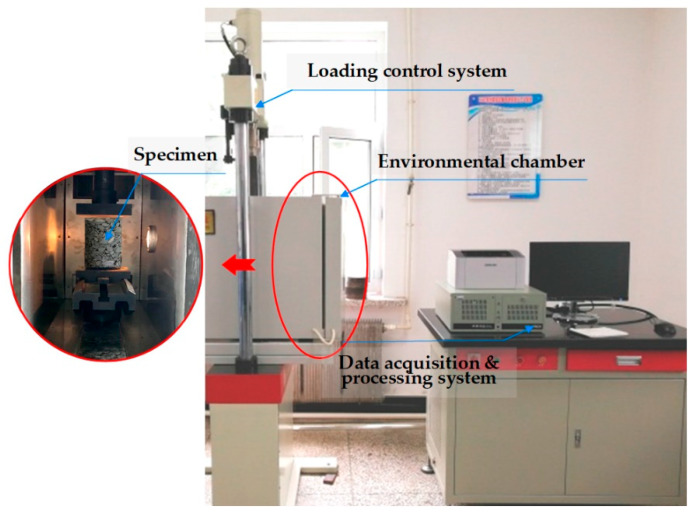
High-temperature uniaxial compression test used in this paper.

The uniaxial compression test is a commonly used test method for determining the compressive strength of asphalt mixtures. In this paper, an electro-hydraulic servo pressure material testing machine produced by Jinli Test Technology Co., Ltd. was selected to perform the uniaxial compression test at 50 °C for basalt fiber-modified asphalt mixtures subjected to F-T cycles. Figure 3 shows the scene of the uniaxial compression test. The testing system includes a loading control system, a data acquisition and processing system, an environmental chamber, and so on. The displacement control was used for loading at a constant strain rate in order to obtain the stress-strain relationship of asphalt mixture specimens. Before the tests, asphalt mixture specimens were kept in the environmental chamber of 50 ± 0.5 °C for more than 4 h; the test loading rate was set to 1 mm/min. Following the standard [30], the compressive strength, failure strain and compression failure stiffness modulus would be determined by the following equations.

*R_c_* = 4*P_c_*/π*d*^2^, (1)

*ε_c_* = Δ*l*/*l*, (2)

S_c_ = R_c_/ε_c_, (3)

where *R_c_* is compressive strength, *P_c_* is the maximum force in compression test, *d* is the diameter of asphalt mixture specimen, Δ*l* is the deformation in the compressive test and *ε_c_* is the compressive failure strain, *S_c_* is compression failure stiffness modulus.

##### Low-Temperature Splitting Test

**Figure 4:**
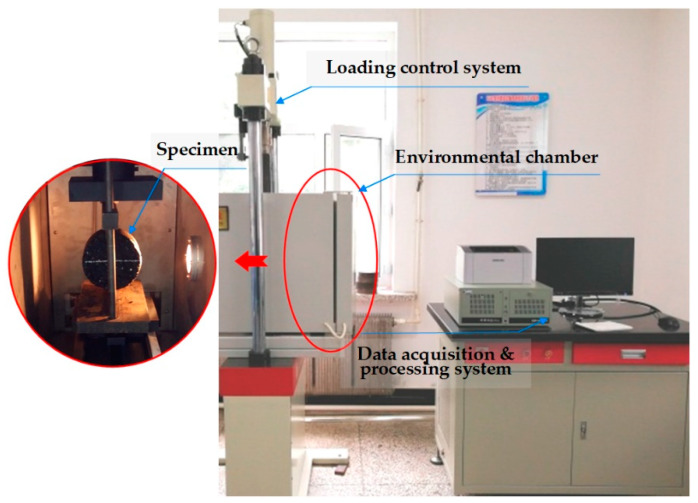
Low-temperature splitting test used in this paper.

Low temperature cracking test is a common and simple test method for evaluating the crack resistance of asphalt mixtures at low temperatures. In this paper, the electro-hydraulic servo pressure material testing machine (Jinli Test Technology Co., Ltd., China) was also selected to perform the low-temperature splitting test at −10 °C for basalt fiber-modified asphalt mixtures subjected to F-T cycles. Figure 4 shows the scene of the low-temperature splitting test. Before the tests, asphalt mixture specimens were kept in the environmental chamber of −10 ± 0.5 °C for at least 6 h. The displacement control was used for loading at a constant strain rate and the test loading rate was also set to 1 mm/min. Following the standard [30], the splitting strength, failure strain and failure stiffness modulus could be calculated by the following equations.

*R_T_* = 0.006287*P_T_*/*h*, (4)

*ε_T_* = *X_T_* × (0.0307 + 0.093*μ*)/(1.35 + 5*μ*), (5)

*S_T_ = P_T_* × (0.27 + 1.0*μ*)/(*h* × *X_T_*)*,* (6)

where *R_T_* is splitting strength, *P_T_* is the maximum force in splitting test, *h* is the height of asphalt mixture specimen, *μ* is the Poisson ratio, *μ* = 0.25 when the test temperature is below 10 °C, *X_T_* is the deformation in the horizontal direction and *ε_T_* is the splitting failure strain, *S_T_* is compression failure stiffness modulus.

##### Indirect Tensile Stiffness Modulus Test

**Figure 5:**
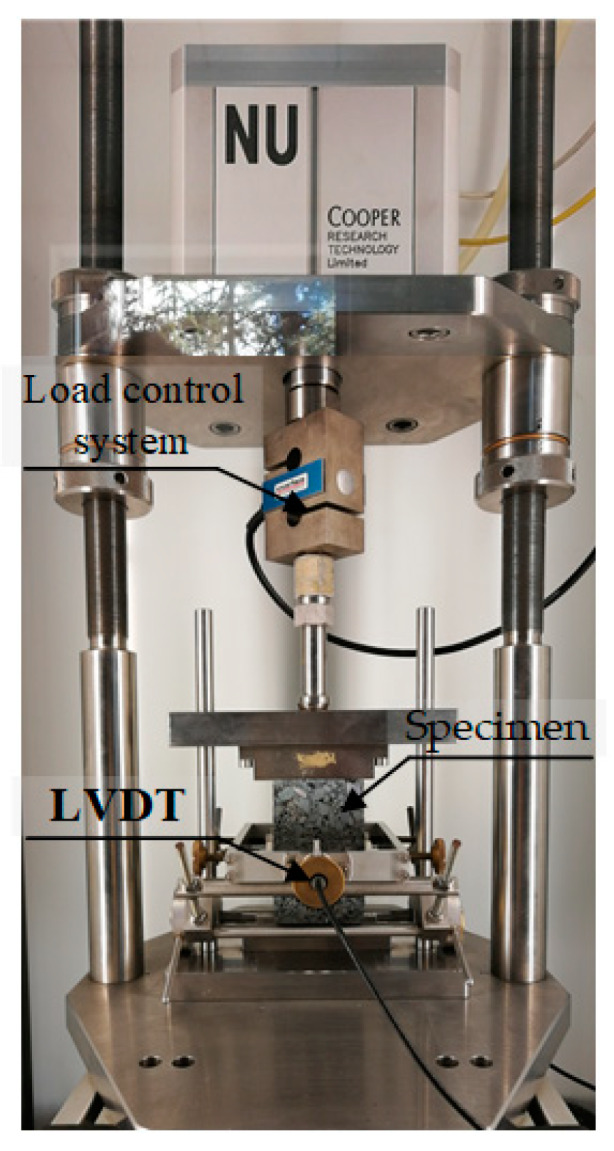
Indirect tensile stiffness modulus (ITSM) test used in this paper.

Dynamic indirect tensile test is usually used to study the tensile properties of asphalt mixtures under dynamic loading. The indirect tensile stiffness modulus (ITSM) can be regarded as an index to evaluate crack resistance. In this paper, according to AASHTO standard, a servo-pneumatic universal testing machine (NU-14, Cooper Technologies Ltd., Ripley, UK) was used to perform the dynamic indirect tensile test of asphalt mixture specimens at 20 °C. The test process is shown in Figure 5. Before the tests, asphalt mixture specimens were kept in the environmental chamber of 20 ± 0.5 °C for at least 5 h. Tor detailed parameters refer to the existing literature [27,29]. Based on the test data, the ITSM (*S_m_*) of asphalt mixture can be obtained through the following equation:

*S_m_ = F* × (*μ* + 0.27)/(*h* × *Z*)*,* (7)

where *F* is the maximum loading, *μ* = 0.35 at 20 °C, *h* is the specimen height, *Z* is the horizontal deformation.

##### Damage Evolution Algorithm and Model Establishment

Based on the reliability and damage theory, a three-dimensional model of freeze–thaw damage evolution of basalt fiber-modified asphalt mixture could be established to investigate the freeze–thaw resistance [31]. According to the above results about compression strength, splitting strength and ITSM, corresponding damage evolution models can be established through damage evolution algorithm. The procedure of damage evolution model of basalt fiber-modified asphalt mixture under F-T cycles are described as:

- Assumption of 3D damage evolution of asphalt mixtures
  (1) The mixtures could be regarded as a continuous homogeneous body. Compared with the specimen size, basalt fiber, aggregates, filler are small enough to disperse uniformly and disorderly in different directions and positions.
  (2) All boundaries of the asphalt mixture are subjected to the same freeze–thaw condition. The microelement points inside asphalt mixture meet the same damage evolution laws.
  (3) The relationship between the F-T damage and the number of F-T cycles of asphalt mixture follows the Weibull distribution as follow:
    *F*(*t*) = 1 − *exp*[−(*λt*)*^α^*]*,* (8)
  (4) The microelement points inside asphalt mixture are affected by the same damage conditions, and the shapes of the failure curve are consistent.
- 3D damage evolution model derivation of basalt fiber-modified asphalt mixtures

The damage probability density function of any microelement point (*x*,*y*,*z*) inside asphalt mixture at time *t* is assumed as *f*(*x*,*y*,*z,t*), the area number of damaged microelement point is *V*(*x*,*y*,*z,t*), satisfying the requirement of spatial Poisson distribution. Then, the damage evolution Equations (9)–(15) could be derived based on the previous study [31]. The probability of the damaged microelement point at time *t* is:

*P* = *f*(*x*,*y*,*z*;*t*)d*ζ*d*η*d*σ*, (9)

The mean of *V*(*x*,*y*,*z,t*) could be obtained from that of Poisson distribution, i.e.,

*E*(*V*) = *nP* = d*x*d*y*d*z*d*ζ*^−1^d*η*^−1^d*σ*^−1^*f*(*x*,*y*,*z*;*t*)d*ζ*d*η*d*σ* = *f*(*x*,*y*,*z*;*t*)d*x*d*y*d*z,* (10)

in which *n* is the number of sample points in the spatial region.

The damaged volume in the entire area is

(11) $$V=\iint_{V_{0}}E(V),$$

in which *V* is the volume of damaged element, *V*_0_ is the volume of original element.

The damage degree (*D*) is defined as

*D* = *V*(*V*_0_)^−1^*,* (12)

By combining the Equations (8) and (10)–(12), the following equation can be deduced.

(13) $$D=V_{0}^{-1}\iint_{V_{0}}f\left(x,y,z;t\right)dxdydz=V_{0}^{-1}\iint_{V_{0}}\alpha {\left(\lambda t\right)}^{\alpha -1}\exp\left[-{\left(\lambda t\right)}^{\alpha }\right]dxdydz,$$

in which *λ* is a scale factor.

In the previous study [31], a detailed description about the damage evolution numerical algorithm has been introduced. Then, the mean of damage degree in the entire area could be finally obtained as:

(14) $$E(D)=E(\omega )/V_{0}=N^{-3}\overset{}{\underset{}{\sum_{i=0}^{N/2-1}\left(6N^{2}-24iN+24i+8\right)}}\left\{1-\exp\left[-{\left(\lambda_{0}t-\frac{ivt}{N/2-1}\right)}^{\alpha }\right]\right\},$$

in which *i* is the number of microelement point, *N* is the division number of each boundary, *ω* is the case of element failure at time *t*.

Then the damage degree after *n* F-T cycles is determined as:

*D_n_* = (*E*_0_ − *E_n_*)/*E*_0_*,* (15)

in which *E*_0_, *E_n_* are the mechanical index of the mixtures after *n* F-T cycles.

## 3. Results and Discussion

### 3.1. High-Temperature Uniaxial Compression under F-T Cycles

**Figure 6:**
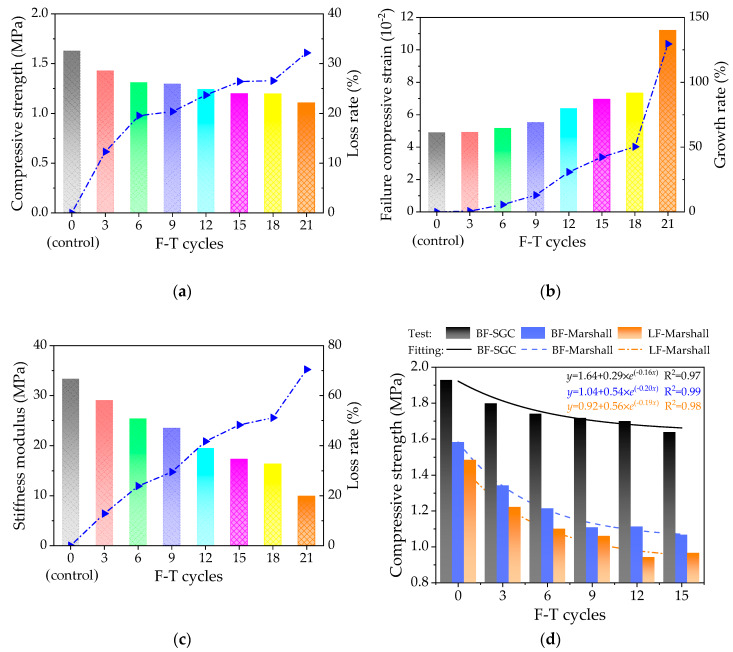
The high-temperature uniaxial compression results: (**a**) compression strength, (**b**) failure strain, (**c**) compression failure stiffness modulus, and (**d**) comparative results.

The effects of F-T cycles on the high-temperature mechanical properties of the asphalt mixtures were characterized by the compressive strength, failure strain and compression failure stiffness modulus, which were obtained and recorded by the Equations (1)–(3). Figure 6 shows the high-temperature uniaxial compression results.

As shown in Figure 6a–c, the histogram plots the compressive strength, failure strain and compression failure stiffness modulus results with F-T cycles. Figure 6a–c show that with F-T cycles increasing, the uniaxial compressive strength and failure stiffness modulus of the asphalt mixtures gradually decrease, while the failure strain increases. Compared to the control group (i.e., unfreeze–thaw asphalt mixture specimen), the dot lines in Figure 6a–c show that the loss rate of compressive strength and failure stiffness modulus increase rapidly then gradually flattened; however, the compressive strength and failure stiffness modulus changed significantly again after 18 F-T cycles. While the growth rate of failure strain shows a gradual increasing trend all the time, the failure strain changed significantly after 18 F-T cycles.

Meanwhile, Figure 6d was plotted based on the previous studies about the performances varying with F-T cycles of the mixtures with basalt fiber and lignin fiber by using Marshall compaction method [32]. It is evident from Figure 6d that the uniaxial compressive strength of different mixtures can be sorted as follows: Basalt fiber-modified asphalt mixture by SGC (BF-SGC) > Basalt fiber-modified asphalt mixture by Marshall compaction method (BF-Marshall) > Lignin fiber-modified asphalt mixture by Marshall compaction method (LF-Marshall). The fitting correlation coefficients (R^2^) show that the exponential function can well reflect the variation trend of uniaxial compressive strength with F-T cycles. The exponential function parameters indicate that the loss rate of compressive strength of BF-SGC is the lowest; the value is 0.16 lower than 0.20 and 0.19.

### 3.2. Low-Temperature Splitting under F-T Cycles

**Figure 7:**
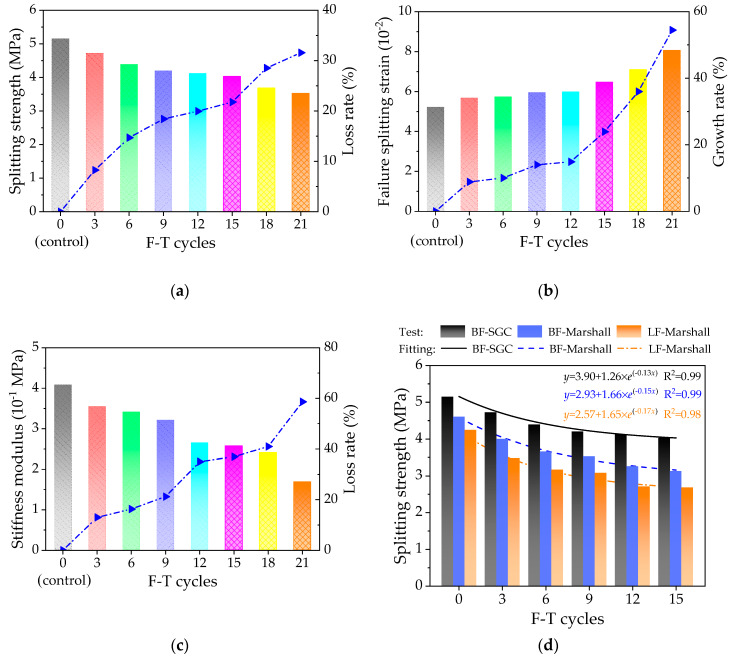
The low-temperature splitting tensile results: (**a**) splitting strength, (**b**) failure strain, and (**c**) splitting failure stiffness modulus, (**d**) comparative results.

The effects of F-T cycles on the low-temperature mechanical properties of the mixtures were characterized by the splitting tensile strength, failure strain and tensile failure stiffness modulus, which were determined and recorded by the Equations (4)–(6). Figure 7 shows the low-temperature splitting results.

As illustrated in Figure 7a–c, the histogram plots the splitting strength, failure strain and splitting failure stiffness modulus results varying with the number of F-T cycles. Figure 7a–c shows that with the increase of F-T cycles, the low-temperature splitting strength and failure stiffness modulus of the mixtures gradually decrease, while the failure strain increases. Compared to the control group (i.e., unfreeze–thaw asphalt mixture specimen), the dot-lines in Figure 7a–c show that the loss rate of splitting strength and failure stiffness modulus are close to S-shaped curve, i.e., the rate increases firstly, then flattens when F-T cycles reach to 12-15, and finally increases. While the growth rate of failure strain shows a gradual increasing trend all the time, and after 12-15 F-T cycles, the failure strain changed significantly.

Similarly, the low-temperature splitting test results were adopted to compare with the splitting performances varying with F-T cycles of the mixtures containing basalt fiber and lignin fiber by using Marshall compaction method in the previous studies [32]. It is clear from Figure 7d that the low-temperature splitting tensile strength of different asphalt mixtures can be sorted as follows: BF-SGC > BF-Marshall > LF-Marshall. The fitting correlation coefficients (R^2^) are all greater than 0.98, which means that the exponential function can well reflect the variation trend of low-temperature splitting tensile strength varying with F-T cycles. The exponential function parameters indicate that the loss rate of splitting tensile strength of BF-SGC is the lowest, the value is 0.13 lower than 0.15 and 0.17. This is because the three-dimensional spatial grid structure formed by adding basalt fibers to asphalt mixture has a reinforcing effect, which can effectively prevent the propagation of crack damage. In addition, asphalt mixtures prepared by SGC had better mechanical performances than those by Marshall compaction method [29].

### 3.3. Indirect Tensile Stiffness Modulus under F-T Cycles

**Figure 8:**
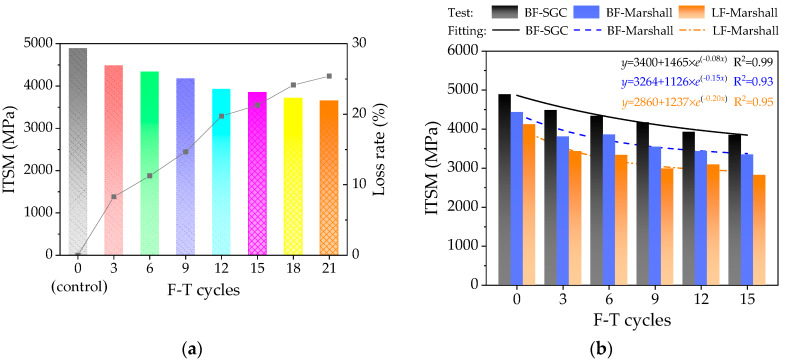
The ITSM results: (**a**) ITSM, (**b**) comparative results.

The influences of F-T cycles on the tensile property under dynamic loading of the mixtures were characterized by the indirect tensile stiffness modulus, which were calculated by the Equation (7). Figure 8 shows the ITSM results.

As shown in Figure 8a, the histogram plots the indirect tensile stiffness modulus results with F-T cycles. Figure 8a describes that ITSM of the mixtures gradually decreases with F-T cycles the increasing. Compared to the control group (i.e., unfreeze–thaw asphalt mixture specimen), the dot-line in Figure 8a shows that the loss rate of ITSM shows a gradual slow increasing trend.

In Figure 8b, the ITSM test results in this paper were used to compare with the ITSM results varying with F-T cycles of the mixtures containing basalt fiber and lignin fiber by using Marshall compaction method in the previous studies [32]. It could be found that the ITSM values of different asphalt mixtures at different F-T cycles would be sorted as: BF-SGC > BF-Marshall > LF-Marshall. Meanwhile, the fitting correlation coefficients (R^2^) are all greater than 0.98, which indicates that the exponential function can well reflect the variation trend of ITSM with F-T cycles. By comparing the exponential function parameters, the exponent parameters can be sorted as BF-SGC (0.08) < BF-Marshall (0.15) < LF-Marshall (0.20), which means that the loss rate of ITSM of BF-SGC is the lowest. Thus, the freeze–thaw resistance of BF-SGC is better than BF-Marshall, and the LF-Marshall specimen has the worse freeze–thaw resistance.

### 3.4. Freeze–Thaw Damage Evolution and Prediction of Basalt Fiber-Modified Asphalt Mixture

From the above mechanical performance results (i.e., *E*_0_ and *E_n_*) under F-T cycles, the damage degree (*D_n_*) can be determined by substituting these results into the Equation (15). Then the damage evolution model would be obtained by the non-linear fitting using F-T cycles t and damage degree. According to the previous study [31], it can be seen that the number of mesh divisions *N* will affect the calculation results. The larger the *N* value, the more accurate the results; however, if the *N* value is too large, it seriously affects the calculation efficiency. In this paper, the number of mesh divisions *N* was determined to be 32 [31]. The damage evolution model was established with the damage degree of 0-12 F-T cycles, and the accuracy of the damage evolution and prediction model was verified with the damage degree results of 15-21 F-T cycles.

The measured mechanical experimental data were adopted to compare with the fitting values of the damage evolution and prediction model to verify its accuracy. Table 1 shows the fitting results of the damage evolution and prediction model parameters *α*, *λ*_0_, and *ν*.

As can be seen from Table 1, the F-T damage evolution model of this kind of mixtures has a higher fitting accuracy, and the correlation coefficient is greater than 0.98. The three model parameters have significant regularity, so this damage evolution and prediction model can be used to analyze the internal degradation law of the mixtures better.

**Figure 9:**
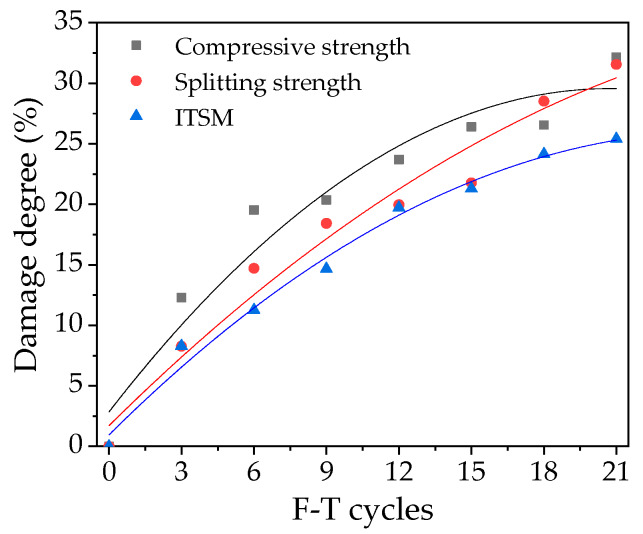
Damage evolution of the asphalt mixture.

The damage evolution law of compression strength, splitting strength and ITSM of the mixtures are shown in Figure 9. Points represent the actual measured damage degree values of the macromechanical performance of mixtures, and the lines are the fitting models. The measured results of 15-21 F-T cycles can be compared with the fitting values. It can be seen from Figure 7 that the error is smaller when fitting the first 12 F-T cycles; the prediction results of 15-21 F-T cycles are close to the actual measurement results. This model can be considered to be more effective and can reflect the evolution law of the mixtures under F-T cycles.

## 4. Conclusions

This paper aimed at the freeze–thaw cycles resistance of SBS modified asphalt mixture reinforced with basalt fiber in order to explore the performance evaluation and prediction of asphalt mixtures under F-T cycles. Asphalt mixture specimens by SGC processed by 0-21 F-T cycles were adopted for the high-temperature compression test, low-temperature splitting test and indirect tensile stiffness modulus test. Conclusions can be achieved as follows:

- The loss rate of compressive strength and failure stiffness modulus increased rapidly, and then gradually flattened, while the growth rate of failure strain showed a gradual increasing trend all the time, and the compressive strength, failure stiffness modulus and failure strain also changed significantly after 18 F-T cycles.
- The loss rate of splitting strength and failure stiffness modulus were close to S-shaped curve, i.e., the rate first increased then flattened when F-T cycles reached to 12-15, and finally increased. While the growth rate of failure strain showed a gradual increasing trend all the time, and after 12-15 F-T cycles, the failure strain changed significantly.
- As for the indirect tensile stiffness modulus, the loss rate of ITSM showed a gradual slow increasing trend. In addition, the exponential function could well reflect the variation trend of the mechanical performances with F-T cycles.
- The damage evolution and prediction model based on the reliability and damage theory can be used to better analyze its internal degradation law.

## Figures and Tables

**Table 1:** Fitting results of damage evolution model parameters.

Mechanical Items	Correlation Coefficient	Model Parameters		
		*α*	*λ*_0_	*ν*
Compressive strength	0.987	0.557	0.057	4 × 10^−8^
Splitting strength	0.988	0.639	0.045	4 × 10^−8^
ITSM	0.991	0.857	0.035	5 × 10^−8^
